# The Citrus Flavanone Naringenin Produces Cardioprotective Effects in Hearts from 1 Year Old Rat, through Activation of mitoBK Channels

**DOI:** 10.3389/fphar.2017.00071

**Published:** 2017-02-27

**Authors:** Lara Testai, Eleonora Da Pozzo, Ilaria Piano, Luisa Pistelli, Claudia Gargini, Maria Cristina Breschi, Alessandra Braca, Claudia Martini, Alma Martelli, Vincenzo Calderone

**Affiliations:** ^1^Department of Pharmacy, University of PisaPisa, Italy; ^2^Interdepartmental Research Center “Nutraceuticals and Food for Health”Pisa, Italy

**Keywords:** naringenin, mitochondrial potassium channels, ischemia/reperfusion injury, cardioprotection, flavonoids, nutraceutics

## Abstract

**Background and Purpose:** Incidence of cardiovascular disorders increases with age, because of a dramatic fall of endogenous self-defense mechanisms and increased vulnerability of myocardium. Conversely, the effectiveness of many cardioprotective drugs is blunted in hearts of 1 year old rat. The Citrus flavanone naringenin (NAR) was reported to promote cardioprotective effects against ischemia/reperfusion (I/R) injury, through the activation of mitochondrial large conductance calcium-activated potassium channel (mitoBK). These effects were observed in young adult rats, but no data are available about the possible cardioprotective effects of NAR in aged animals.

**Experimental Approach:** This study aimed at evaluating the potential cardioprotective effects of NAR against I/R damage in 1 year old rats, and the possible involvement of mitoBK.

**Key Results:** Naringenin protected the hearts of 1 year old rats in both *ex vivo* and *in vivo* I/R protocols. Noteworthy, these effects were antagonized by paxilline, a selective BK-blocker. The cardioprotective effects of NAR were also observed in senescent H9c2 cardiomyoblasts. In isolated mitochondria from hearts of 1 year old, NAR exhibited the typical profile of a mitoBK opener. Finally, Western Blot analysis confirmed a significant (albeit reduced) presence of BK-forming alpha and beta subunits, both in cardiac tissue of 1 year old rats and in senescent H9c2 cells.

**Conclusion and Implications:** This is the first work reporting cardioprotective effects of NAR in 1 year old rats. Although further studies are needed to better understand the whole pathway involved in the NAR-mediated cardioprotection, these preliminary data represent a promising perspective for a rational nutraceutical use of NAR in aging.

## Introduction

Incidence and prevalence of cardiovascular diseases, such as coronary artery disease, hypertension and acute MI, increase proportionally with age; moreover there is a strong correlation between mortality due to MI and age. Indeed, it has been observed that aging reduces cardiac tolerance against the ischemic insult both in human and in animals (Lee et al., 2002; Jahangir et al., 2007; Roger et al., 2011; Wojtovich et al., 2012). In aged myocardium, endogenous self-defense mechanisms, such as “ischemic pre-conditioning” (IPC), are less effective and, consistently, the vulnerability of the heart is increased (Boengler et al., 2009). The number of elderly people is growing and in 2020 at least one-fifth of the population is predicted to be composed of over 65 individuals; therefore the understanding of the mechanisms which regulate cardiac senescence and the development of effective pharmacological strategies for myocardial protection is an urgent issue.

The fundamental processes that lead to cardiac aging and loss of myocardial resistance are not yet completely understood. Mitochondrial dysfunction in electron transport or in oxidative phosphorylation is closely implicated in the increased susceptibility to ischemic injury. These defects witnessed by partial depolarization of membrane potential of aged mitochondria and higher probability of MPTP opening (Navarro and Boveris, 2007).

The activation of mitoKATP is a pivotal step in the endogenous mechanisms of cardioprotection against I/R (Testai et al., 2015). In aging, such mitoKATP-mediated cardioprotective effects are compromised (Liu et al., 2011), but it is still unclear whether this is due to changes in channel density, decreased responsiveness to activating stimuli or defective respiratory chain (Tricarico and Camerino, 1994; Tani et al., 2001).

Recent data of ours (Testai et al., 2013a,b) suggested that NAR, a flavanone abundant in the Citrus genus, besides already known vasoactive properties (Calderone et al., 2004; Saponara et al., 2006), is able to promote cardioprotective effects in young-adult rats (3–4 months old). These effects may be due, at least in part, to anti-oxidant properties typical of flavonoids; however, specific effects of flavonoids on mitochondria- such as potassium influx, decreased calcium uptake into the matrix, and a mild mitochondrial membrane depolarization – may play a relevant role. These effects are likely to be due to mitoBK activation (Testai et al., 2013b, 2015; Testai, 2015).

This study aimed at evaluating, in 1 year old rats, the potential cardioprotective effects of NAR against I/R damage, and the possible involvement of mitoBK in its mechanism of action.

## Materials and Methods

### Substances

Naringenin was purchased from Sigma–Aldrich (CAS Number 67604-48-2, >95% of purity). The flavonoid was dissolved (10^-2^ M) in DMSO, and further diluted in bi-distilled water. PAX and valinomycin were purchased from Sigma–Aldrich (Milano, Italy), dissolved in DMSO (10^-2^ M) and further diluted in bi-distilled water. TPP^+^Cl^-^ (Sigma–Aldrich, Milano, Italy) was dissolved in bi-distilled water and TTC (Sigma–Aldrich. Milano, Italy) was dissolved (1%, p/w) in phosphate buffer (pH 7.4).

### Pharmacological Procedures

All the experimental procedures were carried out following the guidelines of the European Community Council Directive 2010/63 and have been approved by the Animal Care Committee of the University of Pisa, Italy (Protocol. N. 37321, October 22nd, 2013).

#### Langendorff-Perfused Rat Hearts

One year old male Wistar rats (400–500 g) were treated intraperitoneally with NAR (100 mg/kg) or DIAZO, (40mg/Kg) or their vehicle (DMSO). After 2 h, all the animals were anesthetized with sodium pentobarbital (100 mg/kg i.p.) and heparinized (100 UI i.p.); hearts were quickly excised and mounted on a Langendorff apparatus, as previously described by Calderone et al. (2010) and Testai et al. (2013a).

Heart rate, LVDP, and dP/dt were continuously monitored by a computerized Biopac system Inc. (Goleta, CA, USA). The functional parameter of RPP was calculated as RPP = HR × LVDP. After 30 min of equilibration (pre-ischemic period), the hearts were subjected to 30 min of global ischemia (no flow). At the end of the ischemic period the hearts were re-perfused for a period of 120 min. At the end of the reperfusion period, left ventricle was cut in 2 mm large slices which were immersed in a 1% aqueous solution of TTC for 20 min and then in a 10% aqueous solution of formaldehyde. After 24 h, the ventricular slices were photographed and analyzed in order to highlight the necrotic areas due to the ischemic process (visible as a white or light pink color and areas) and the healthy areas (visible as strong red areas due to the TTC reaction).

##### Data analysis

In order to evaluate the cardiac inotropism in the reperfusion phase, the LVDP recorded in the reperfusion time was calculated and expressed as a percentage of the pre-ischemic value, recorded at the last minute of perfusion. Furthermore, the ischemic areas were evaluated planimetrically and expressed as a percentage of the whole area of the slices of the left ventricle (Ai/*A*_LV_). All the values are expressed as a mean ± standard error for 6–8 different experiments. Data were statistically analyzed by ANOVA and *P*-values lower than 0.05 were considered as indicative of significant differences.

#### *In vivo* Myocardial Acute Infarct

Two hours before the experimental procedures, 1 year old rats (400–500 g) received an i.p. injection (about 0.5 ml) of NAR (100 mg/kg) or vehicle (DMSO).

Then, rats were anesthetized with sodium pentobarbital (70 mg/kg, i.p.) and the experimental protocol for coronary occlusion-reperfusion was performed as described in Calderone et al. (2010) and Testai et al. (2013b).

The acute infarct protocol consisted of 30 min occlusion/120 min reperfusion; successful occlusion was confirmed by observing regional cyanosis downstream of the ligature, and by ST elevation in the ECG recording. In another experimental group, the selective BK-blocker PAX was administered (10 mg/kg i.p.) 10 min before the administration of NAR. A group of vehicle-pretreated animals was submitted to an IPC procedure, achieved by two cycles of 5 min occlusion/10 min reperfusion, followed by 30 min coronary occlusion and 120 min reperfusion. At the end of reperfusion, rats were euthanized by an overdose of sodium pentobarbital, then hearts were quickly excised, mounted on a Langendorff apparatus (Radnoti, Monrovia, CA, USA) and perfused for 10 min with Krebs solution at 37°C to wash out the coronary blood vessels. Then, left ventricular tissue was dried, frozen at -20°C, and cut into 4–5 transverse slices from the apex to the base of equal thickness (about 2 mm). The slices were then incubated in a TTC solution in a phosphate buffer (pH 7.4) at 37°C for 20 min. TTC reacts with NADH in the presence of dehydrogenase enzymes, to form a formazan derivative, which stain the viable cells with intense red color. Then, the slices were fixed overnight in 10% formaldehyde and finally they were photographed. In the viable area, red-stained viable tissue was easily distinguished from the white-unstained necrotic tissue.

##### Data analysis

The Ai was planimetrically evaluated using an image analyzer program (The GIMP 2). The infarct size was calculated as a percentage of the whole left ventricle area (Ai/*A*_LV_).

All the values are expressed as a mean ± standard error for 6–8 different experiments. Data were statistically analyzed by ANOVA and *P*-values lower than 0.05 were considered as indicative of significant differences.

#### Isolated Cardiac Mitochondria

##### Isolation procedure

Rat cardiac mitochondria were isolated by differential centrifugation, as previously described (Calderone et al., 2010; Testai et al., 2013b, 2016), with minor modifications. 1 year old male Wistar rats (400–500 g) were killed by pentobarbital overdose, hearts were removed immediately and placed in an ice cold isolation buffer (IB1, composition mM: sucrose 250, Tris 5, EGTA 1, pH 7.4 adjusted with HCl). The atria were removed and the ventricular tissue was finely minced with surgical scissors (about 2 mm^3^pieces) and homogenized using an Ultra-Turrax homogenizer (20 ml of isolation buffer per heart, IKA1-Werke GmbH & Co., Staufen, Germany).

Three homogenization cycles (each of 20 s) were performed on ice, and then the suspension was centrifuged at 1075 *g* for 3 min at 4°C (EuroClone, Speed Master 14 R centrifuge, Milano, Italy). The resulting supernatant was centrifuged at 11950 *g* for 10 min at 4°C. The pellet containing the mitochondrial fraction was further re-suspended in the isolation buffer (without EGTA, IB2) and centrifuged at 11950 *g* for 10 min at 4°C, this step was repeated once more. The final mitochondrial pellet was re-suspended in a minimal volume of 400 μl of the IB2 and stored on ice throughout the experiments, which were performed within 2 h. Mitochondrial protein concentrations were determined using the usual Bradford reaction.

##### Mitochondrial membrane potential

Mitochondrial membrane potential (Ψm) was potentiometrically measured with tetraphenylphosphonium (TPP^+^)-sensitive mini-electrodes, coupled with a reference electrode (WPI, FL, USA), using a data acquisition software (Biopac Systems Inc., Goleta, CA, USA), as previously described (Calderone et al., 2010). Briefly, electrodes were calibrated before each experiment using known concentrations of TPP^+^Cl^-^. Mitochondria (1 mg protein/ml) were suspended under gently stirring in the IM, (composition mM: KCl 120, K_2_HPO_4_ 5, Hepes 10, succinic acid 10, MgCl_2_ 2, EGTA 1, plusTPP^+^Cl^-^10 μM, pH 7.4 adjusted with KOH). The potential value was calculated by a Nernst-derived experimental equation as published by Lábajová et al. (2006). Changes of Ψm were continuously recorded (in mV) before and after the addition in the IM of cumulative increasing concentrations of NAR (10–100 μM). When required, PAX (10 μM), a blocker of BK channels, was incubated in the medium 2 min before the mitochondria addition. Moreover, the effects of the addition of the corresponding vehicle were evaluated.

###### Data analysis

The effects of NAR on the mitochondrial membrane potential were expressed as changes (in mV) from the basal levels. All data were expressed as a mean ± standard error. Each concentration-response curve was obtained with mitochondria isolated from the hearts of 6–10 different animals. Data were statistically analyzed by ANOVA and Student’s *t*-test (software: GraphPad Prism 5.0). *P*-values lower than 0.05 were considered as indicative of significant differences.

##### Mitochondrial calcium-uptake

Mitochondrial calcium-uptake was measured by potentiometric technique, as previously described (Calderone et al., 2010). In particular, the changes of the calcium concentration in the medium (i.e., extra-mitochondrial calcium) were continuously measured with a calcium-selective mini-electrode, coupled with a reference electrode (WPI, FL, USA), using a data acquisition software (Biopac Systems Inc., Goleta, CA, USA). In order to correlate the potentiometric measurements (in mV) with the corresponding concentrations of calcium ions in the solution, calibration curves were generated before each experiment, by using known concentrations of CaCl_2_. Mitochondria (1 mg protein/ml) were added, under gently stirring, to the IM plus CaCl_2_ 100 μM in the presence of vehicle (DMSO 1%) or NAR (100 μM). After the addition of mitochondria, the maximal decrease of the calcium concentration in the medium, related to its accumulation in the mitochondrial matrix, was measured.

###### Data analysis

Mitochondrial Ca^2+^-uptake was evaluated by measuring the reduction of the extra-mitochondrial Ca^2+^ concentration, following the addiction of mitochondria into calcium rich buffer. All data are expressed as a mean ± standard error. Data were statistically analyzed by ANOVA and Student’s *t*-test (software: GraphPad Prism 4.0). *P*-values lower than 0.05 were considered as indicative of significant differences. Each result was obtained with mitochondria isolated from the hearts of 3–6 different animals.

##### Activation of mitochondrial potassium channels

The potential NAR-induced activation of mitochondrial potassium channels was carried out with a novel fluorimetric approach, based on the potassium-mimetic behavior exhibited by thallium ions (Tl^+^) and the use of a specific Tl^+^-sensitive fluorescent probe (benzothiazole coumarin acetoxymethyl ester), available in a commercial kit (FluxOR, Invitrogen, Monza, Italy). Such an approach, originally used to study sarcolemmal potassium channels, has been set up to evaluate the modulation of potassium channels on isolated mitochondria (Testai et al., 2013b). After the above isolation procedures, mitochondria were incubated with loading buffer (containing the Tl^+^-sensitive probe) for 10 min at room temperature under continuous stirring in the dark, in order to allow the Tl^+^- sensitive probe to accumulate into the mitochondrial matrix. Then, the mitochondria were diluted with IB2 and centrifuged at 11,950 *g* for 10 min at 4°C, this step was repeated twice, in order to remove the residual Tl^+^-sensitive probe from the extramitochondrial medium. The final pellet was re-suspended in a minimal volume (400 μl) of IB2 and stored on ice until use. Mitochondrial proteins were determined via Bradford method.

Immediately before the test, the stored mitochondrial suspension was further diluted (0.5 mg mitochondrial protein/ml) in mannitol buffer (composition: mannitol 240 mM, Na_2_HPO_4_ 5 mM, Hepes 10 mM, succinic acid 10 mM, MgSO_4_ 2 mM, ATP 200 μM, pH 7.4). Then, into a black 96-multiwell plate (80 μl/well) suitable for the fluorescence assay, the potassium ionophore valinomycin (2 μM, reference compound), NAR (100 μM), or their corresponding vehicle (DMSO 1%) were added in the wells and finally, after 2 min, aqueous solution of Tl_2_SO_4_ (100 μM) was further added into each well.

###### Data analysis

The increase in fluorescence (due to the eventual Tl^+^ entry into the matrix, through potassium channels) was monitored (λex = 488 nm, λem = 525 nm) by the EnSpire multiplate reader (PerkinElmer, Boston, MA, USA). These experiments were completed within 1.5 h after the isolation of mitochondria. Each result was obtained with mitochondria isolated from the hearts of 3–6 different animals. All data were expressed as a mean ± standard error. Data were statistically analyzed by ANOVA and Student’s *t*-test (software: GraphPad Prism 5.0). *P*-values lower than 0.05 were considered as indicative of significant differences.

#### Cell Cultures

H9c2 subclonal cell line, deriving from embryonic rat hearts (Hescheler et al., 1991) was purchased from ATTC (Manassas, VA, USA). H9c2 cells were cultured, following the usual procedures in DMEM, (Sigma–Aldrich, St. Louis, MO, USA) supplemented with 10% FBS, (Sigma–Aldrich, St. Louis, MO, USA), 100 units/ml penicillin and 100 mg/ml streptomycin in tissue culture flasks at 37°C in a humidified atmosphere of 5% CO_2_.

##### H9c2 Cell Senescence model

Senescence in H9c2 cells was induced by Dox as previously reported (Spallarossa et al., 2009). Briefly, cells were cultured up to about 80% confluence in a DMEM medium and before the experiments, cells were seeded onto 24-well plates at a density of 10 × 10^3^ cell/cm^2^. After 24 h to allow cell attachment, the medium was replaced in each well and the cells received different treatments such as vehicle (0.1% DMSO), various concentrations of Dox (0.01, 0.05, and 0.1 μM) for 3 h, and were subsequently cultured for 3 days, after which senescence was determined.

###### Senescence-associated β-galactosidase (sa-beta-Gal) staining

To evaluate the number of senescent cells after 3 days from Dox insult, the senescence marker sa-beta-Gal was detected as previously reported (Costa et al., 2013). According to the method described, treated cells were fixed in p-formaldehyde and incubated in freshly prepared staining solution for 16 h at 37°C in a dry incubator. Cells were then washed in PBS (1×) and photographed at 100× magnification. Images of random light microscopic fields were captured (five fields per well), and both blue and total cells were counted using ImageJ (ImageJ Software, version 1.41; USA).

###### RNA extraction and Real Time PCR analysis

After 72 h Dox treatment, injured H9c2 cells were collected, and total RNA was extracted using RNeasy^®^ Mini Kit (Qiagen, Hilden, Germany) according to the manufacturer’s instructions. cDNA synthesis was performed with 500 ng of RNA using the i-Script cDNA synthesis kit (Bio-Rad, Hercules, CA, USA) following the manufacturer’s instructions. Real time RT-PCR reactions consisted of 25 μL of Fluocycle^®^ II SYBR^®^ (Euroclone, Milan, Italy), 1.5 μL of both 10 μM forward and reverse primers, 3 μL of cDNA, and 19 μL of H_2_O. All reactions were performed for 38 cycles using the following temperature profiles: 94°C for 1 min (initial denaturation); 55–59°C for 30 s (annealing); and 72°C for 1 s (extension).

###### Cell cycle analysis

The measurement of the percentage of Dox injured cells in the different cell phases was performed using the Muse^TM^ Cell Analyser (Merck KGaA, Darmstadt, Germany). Briefly, senescent adherent cells were collected and centrifuged at 300 × *g* for 5 min. The pellet was washed with PBS and suspended in 100 μl of PBS; finally, the cells were slowly added to 1 ml of ice cold 70% ethanol and maintained over night at -20°C. Then, a cell suspension aliquot (containing at least 2 × 10^5^ cells) was centrifuged at 300 × *g* for 5 min, washed once with PBS and suspended in the fluorescent reagent (Muse^TM^ Cell Cycle reagent) (Daniele et al., 2014).

###### Data analyses

The non-linear multipurpose curve-fitting program Graph-Pad Prism (GraphPad Software Inc., San Diego, CA, USA) was used for data analysis and graphic presentations. All results are presented as the means ± standard errors of the means (SEM) of data for duplicate samples and are representative of three different experiments. Statistical analysis was performed by one-way analysis of variance (ANOVA) with Bonferroni’s corrected *t*-test for *post hoc* pair-wise comparisons, or by *t*-test. *P* < 0.05 was considered statistically significant.

##### Cell viability on anoxia/reoxygenation (A/R) injury

To simulate anoxia in senescent H9c2 cells, plated cells (3.5 × 10^3^ cells/cm^2^) were exposed to a low-glucose and serum-free solution, added with NAR (4–40 μM) in the presence or in the absence of PAX (10 μM), and sealed for 16 h in airtight containers saturated with 95% N_2_ and 5% CO_2_ (37°C), whereas twin plates were placed in 95% air and 5% CO_2_ (normoxic conditions), as previously reported (Calderone et al., 2010). Then, cells were re-oxygenated for 2 h in normoxic conditions (37°C). After re-oxygenation, cell viability was assessed by quantitative colorimetric MTS assay kit according to manufacturer (Cell Titer 96^®^ AQueous one Solution Cell Proliferation assay, Promega). After 2 h of incubation with the reagent, the absorbance of individual wells was measured by microplate reader (Wallac Victor 2, 1420 Multilabel counter, PerkinElmer). Each drug concentration was tested in triplicate and the experiments were repeated at least three times.

#### Western Blot Analysis

The mitochondrial pellet obtained from rat ventricles and H9c2 cells were resuspended in IB2. When mitochondrial pellet from left ventricular tissue was used, rat hearts were obtained from young (3–4 months old) and 1 year old animals. 100 μl of mitochondrial suspension were incubated with 2.5 μl of Triton X-100 20%, 10 μl KCl 2.5 M, 1 μl of protease inhibitor cocktails and incubated at room temperature for 5 min. After centrifugation (12.000 rpm for 5 min) the protein concentration was determined by Bradford (Bio-Rad, Milan, Italy) method. The samples were preserved by adding an equal volume of loading buffer (1:1) (Sample Buffer, Sigma–Aldrich, Milan, Italy).

Fifty micrograms of mitochondrial proteins were loaded on 4–20% sodium dodecyl sulfate–polyacrylamide precast gel (cod. 4561095, Bio-Rad, Milan, Italy), separated by electrophoresis and transferred to a polyvinylidene fluoride membrane by Semi-Dry Electrophoretic transfer cell for 7 min a 12,5V (cod. 1703940, Bio-Rad, Milan, Italy).

Unspecific binding of the antibody was blocked by incubation with 3% fat-free milk solution in Tris-buffered saline containing 0.1% Tween for 1 h. Subsequently, the membrane was incubated overnight at 4°C with anti-MaxiKa or anti-Sloβ1 subunit antibodies (1:1,000 and 1:200, respectively, AbCam, Cambridge, UK). After washing (3 × 10 min) in 3% fat-free milk solution, the blot was subjected to the appropriate horseradish peroxidase – conjugated secondary antibody for 2 h at room temperature.

##### Data analysis

Bands were visualized using a chemoluminescence kit (Millipore, Milan, Italy) and quantified by optical densitometry. Images were acquired using Image Quant LAS4010 (GE Healthcare, Buckinghamshire, UK). On the same blots, protein contents were normalized to the amounts of ATP5A (1:500, AbCam, Cambridge, UK).

## Results

### Effects on Isolated Heart Submitted to I/R

**FIGURE 1:**
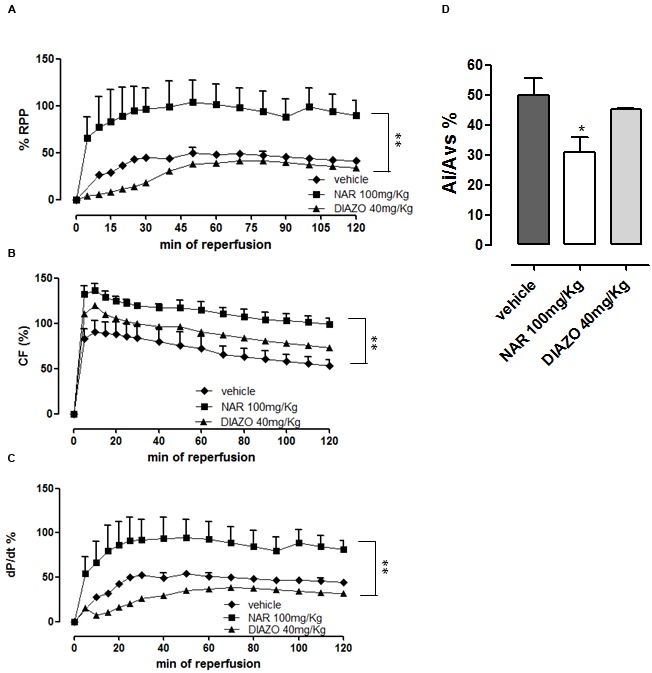
**Post-ischemic functional parameters of RPP (A)**, CF **(B)**, and dP/dt **(C)** recorded during the reperfusion time in Langendorff-perfused hearts of 1 year old rats pre-treated with vehicle, NAR 100 mg/kg or DIAZO 40 mg/kg. Two way ANOVA indicated that the curves obtained following NAR-treatment show significant differences (*P* < 0.01) vs. vehicle. The corresponding data emerging from the morphometric analysis of the tissue injury are also shown; asterisks indicate significant differences (^∗^*P* < 0.05) vs. vehicle **(D)**.

Ischemia/reperfusion episode caused an evident myocardial damage in isolated hearts of 1 year old rats. Indeed, when compared with the corresponding pre-ischemic values, functional parameters of RPP and dP/dt were about half-reduced during the whole reperfusion period; moreover the histological assay highlighted an extended damage area (Ai/*A*_LV_ = 50 ± 6%) which was significantly higher than that observed in the young one (Ai/*A*_LV_ 36 ± 7%) (**Figures 1A–D**).

In isolated hearts of 1 year old rats, DIAZO did not produce cardioprotective effects and the post-ischemic functional and histological parameters were almost identical to the control ones (RPP value was superimposable with vehicle and Ai/*A*_LV_ was 45 ± 1%) (**Figures 1A–D**).

Surprisingly, NAR was cardioprotective in hearts from 1 year old rats submitted to I/R episode. Indeed, functional recovery was significantly improved (RPP% 90 ± 16 at 120th min of reperfusion) and it was quite stable for the whole reperfusion period (**Figure 1A**). In agreement with the inotropic value, ischemic area size was markedly reduced (Ai/*A*_LV_% = 31 ± 5) (**Figure 1D**).

### Effects of NAR in Acute Myocardial Infarct

**FIGURE 2:**
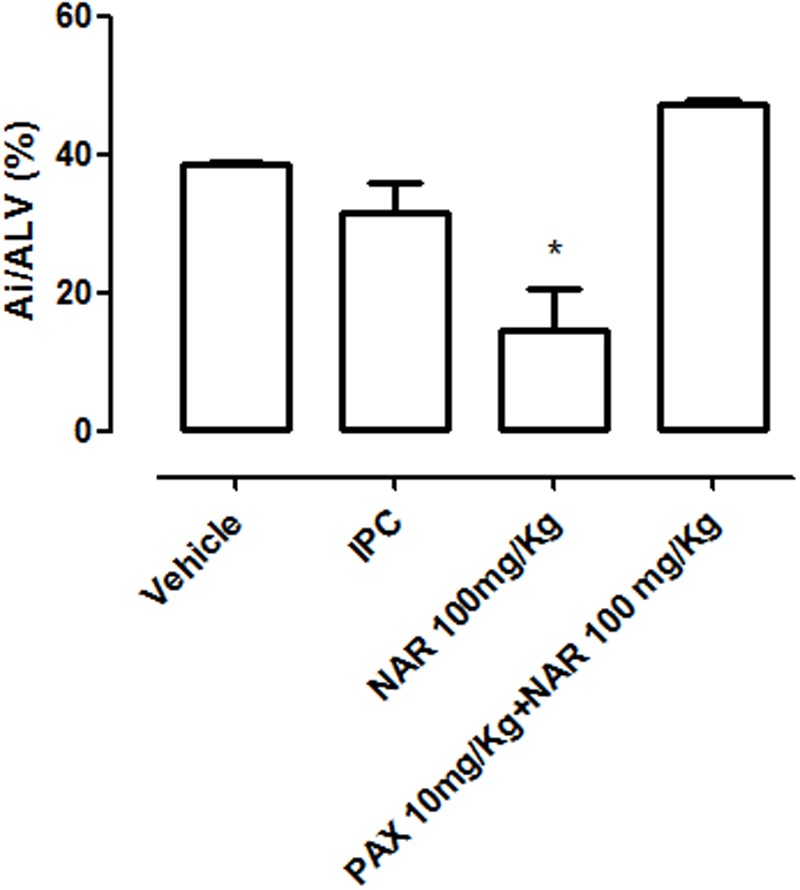
**Values of injured areas (expressed as % of the left ventricle area Ai/*A*_LV_ %) observed in the left ventricles isolated from 1 year old rats treated with vehicle, NAR, NAR+PAX or submitted to pre-conditioning ischemia (IPC) before the protocol of *in vivo* acute infarct.** The asterisks indicate a statistically significant difference from the value observed in the hearts of vehicle treated animals (^∗^*P* < 0.05).

In vehicle-treated 1 year old animals, submitted to the experimental protocol of acute infarct, large extension of I/R injury could be detected (Ai/*A*_LV_ = 38 ± 0.5%). IPC induced no significant reduction of the I/R injury (Ai/*A*_LV_ = 32 ± 4%), whereas the pharmacological pretreatment with NAR led to a significant reduction of the I/R injured areas (Ai/*A*_LV_ = 15 ± 6%). PAX completely abolished the cardioprotective effects of NAR (Ai/*A*_L_ = 47 ± 1%) (**Figure 2**).

### Mitochondrial Effects of NAR

**FIGURE 3:**
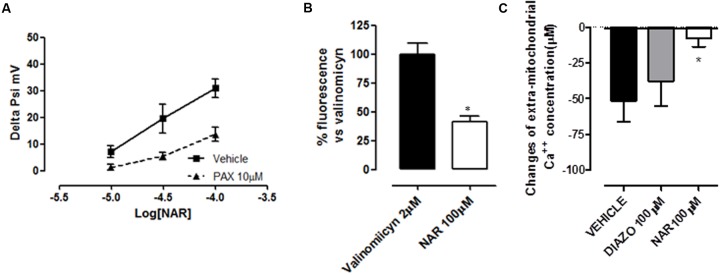
**(A)** Concentration-dependent changes in mitochondrial membrane potential, induced by the cumulative administration of increasing concentrations of NAR to isolated cardiac 1 year old mitochondria. Two-way ANOVA indicated statistically significant differences between the curve obtained in control condition and those obtained in the presence of PAX (10 μM). **(B)** Increase of the fluorescence (expressed as a % of that induced by the potassium-ionophore valinomycin) produced by a Tl^+^-sensitive probe accumulated into the mitochondrial matrix, after the addition of Tl^+^ ions to a suspension of isolated cardiac 1 year old mitochondria in the presence of NAR (100 μM). The asterisks indicate a statistically significant difference (^∗^*P* < 0.05). **(C)** The reduction of the extra-mitochondrial calcium concentration (in μM), following the administration of isolated cardiac mitochondria from 1 year old rats in the presence of NAR 100 μM, DIAZO 100 μM, or their vehicle. The asterisks indicate a statistically significant difference (^∗^*P* < 0.05).

Cardiac mitochondria from 1 year old rats possess a membrane potential slightly but significantly more depolarized if compared with young ones, in agreement with literature (-159 ± 6 vs. -174 ± 4 mV; Jahangir et al., 2001). The addition of cumulatively increasing concentrations of NAR (10–100 μM) to a suspension of cardiac mitochondria from 1 year old rats (1 mg/ml) caused a concentration-dependent depolarization of the mitochondrial membrane, with a maximum increase of 31 ± 4 mV caused by the highest concentration of the flavonoid. Moreover, NAR-induced effects were significantly antagonized by PAX 10 μM (**Figure 3A**). In contrast, DIAZO, administrated to cardiac mitochondrial suspensions deriving from 1 year old rats, didn’t produce significant effects on membrane potential (data not shown). The addition of the potassium ionophore valinomycin (2 μM) to isolated cardiac mitochondria allowed the potassium-mimetic Tl^+^ ions to enter massively into the mitochondrial matrix. Such an effect was witnessed by great increase of fluorescence of the Tl^+^-sensitive probe previously accumulated into the matrix.

The administration of NAR 100 μM led to a significant increase in fluorescence (41 ± 5% of the fluorescence triggered by valinomycin), indicating a large Tl^+^ influx into the matrix (**Figure 3B**).

Moreover, cardiac mitochondria derived from 1 year old rats were able to accumulate calcium into the matrix (51 ± 15μM). However, the calcium up-take in the mitochondria of 1 year old hearts was about half reduced, when compared with that observed in young ones (data not shown). As expected, DIAZO 100 μM was ineffective in 1 year old cardiac mitochondria (calcium up-take: 38 ± 18 μM). In contrast, NAR significantly inhibited the calcium up-take in 1 year old rat-derived mitochondria (8 ± 6 μM) (**Figure 3C**).

#### Induction of H9c2 Cell Senescence

**FIGURE 4:**
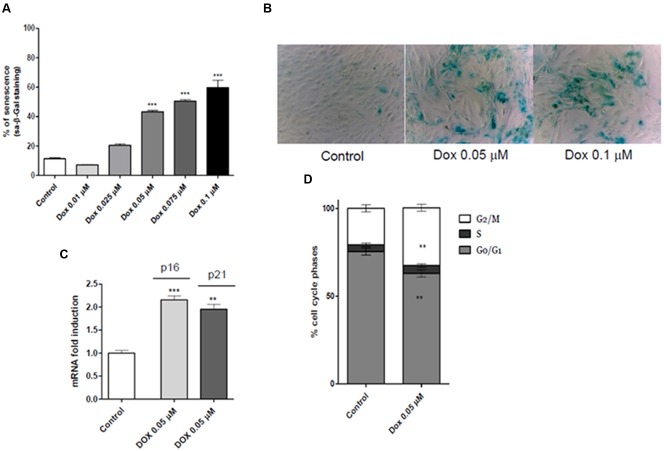
**(A)** Percentage of β-galactosidase positive cells observed after treatment with different increasing concentrations of Dox. **(B)** Representative phase contrast photomicrographs of treated cells (control, Dox 0.05 μM and Dox 0.1 μM, respectively). **(C)** Real mRNA levels, indicative of an induction of p16 and p21 mRNA, and **(D)** cell cycle phases analyses. The asterisks indicate a statistically significant difference (^∗∗∗^*p* < 0.001, ^∗∗^*p* < 0.01 vs. control group).

A concentration-dependent increase of sa-β-gal was observed following the H9c2 cell treatment with increasing concentrations of Dox (**Figures 4A,B**). These results allowed us to establish Dox 0.05 μM as the effective concentration for the following H9c2 cell experiments.

Moreover, the real time RT-PCR results showed that Dox cell treatments significantly increased the p16 and p21 mRNA levels in H9c2 cells (*p* < 0.01 and *p* < 0.001, **Figure 4C**). Consistent with these results, cell cycle analysis evidenced arrest of cell cycle progression in G2/M phase after Dox treatments (*p* < 0.01 and *p* < 0.001, **Figure 4D**).

#### A/R Survival of H9c2 Cells

**FIGURE 5:**
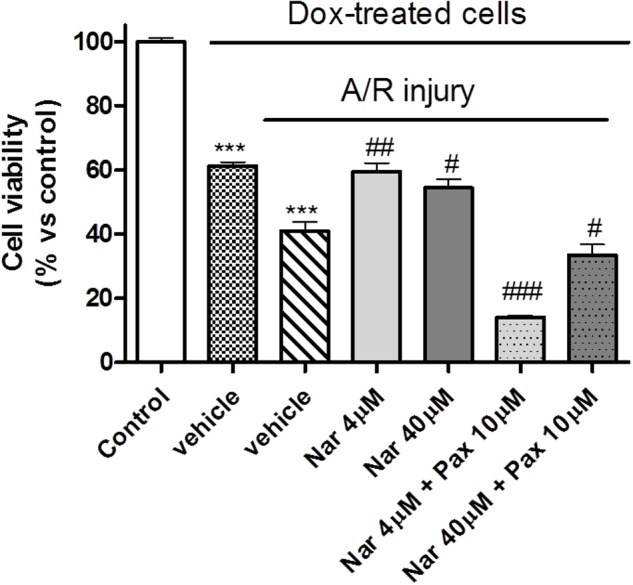
**Cell viability of senescent H9c2 cells under hypoxic conditions.** Percentage of cell viability compared to the control (non-senescent normoxic H9c2 cells) to which a viability of 100% was attributed. The asterisks indicate a statistically significant difference vs. control (^∗∗∗^*p* < 0.001); the hashtags indicate statistically significant difference vs. A/R injury vehicle. (^###^*p* < 0.001, ^##^*p* < 0.01, ^#^*p* < 0.05).

In normoxic conditions, Dox-mediated induction of cell senescence was associated with a significant reduction in cell viability, with respect to non-senescent control cells (about 60%, *P* < 0.01). When senescent H9c2 cells were subjected to A/R a further reduction of cell viability was recorded (40%, *p* < 0.001). NAR (4 and 40 μM) evoked significant cytoprotective effects against A/R injury, resulting in a significant improvement of cell viability (about 60 and 55%, respectively); the BK-blocker PAX 10 μM markedly antagonized the protective effects of NAR (**Figure 5**).

### Western Blot Detection of mitoBK in Tissue and Cells

**FIGURE 6:**
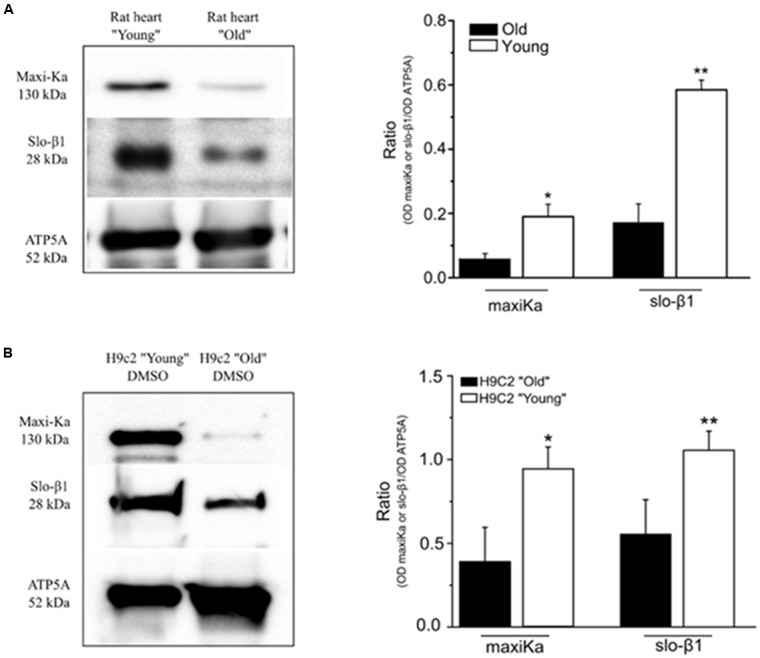
**Reduction of α and β subunits of BK channel, indicated as maxiKa and slo-β1, respectively, in mitochondrial membranes. (A)** Western blot analysis of both α and β subunits in mitochondrial membranes obtained from young and old rat ventricle. Left panel shows a representative example of western blot experiment. Right panel shows a significant reduction of protein expression in “old” ventricle (black bars) respect to “young” ventricle (white bars). **(B)** Western blot analysis of both α and β subunits in mitochondrial membranes obtained from H9c2 cell line after treatment with Dox (“old” cell) or DMSO alone (“young” cell). Left panel shows a representative example of western blot experiment. Right panel shows a significant reduction of protein expression in “old” H9c2 (black bar) compared to the “young” H9c2 (white bars). Bars represent the mean ± SEM (*n* = 3 independent experiments). Statistic *t*-test ^∗^*P* < 0.05, ^∗∗^*P* < 0.01.

Western blot analysis, using α- and β1-subunit-specific antibodies, was carried out in mitochondrial lysate from ventricle tissue of young and 1 year old rats, as well as in “young” and “aged” H9c2 cardiomyoblasts. It detected bands of 130 and 28 kDa, respectively (**Figure 6**). Immunoblots showed that both α- and β1-subunit are present in mitochondria of young and 1 year old hearts, as well as in “young” and “aged” H9c2 cells. However, protein expression dramatically decreased with aging, in both the experimental models. The relative amount of α- and β1-subunit of the BK channel (normalized to the ATP5A signal in three different membranes) is shown in **Figure 6A** for mitochondrial lysate obtained from rat ventricle, and in **Figure 6B** for mitochondrial lysate obtained from H9c2 cell line.

## Discussion

During dramatic cell stress, such as I/R, mitochondria play a crucial role in deciding the cell fate: apoptotic death or survival. Mitochondria are very sensible to oxidative damage, naturally associated with aging. Indeed, the oxidative stress accounts for defective mitochondrial protein synthesis, alteration in the mitochondrial respiratory chain and loss of mitochondrial membrane potential, influencing the calcium homeostasis and the mitochondrial bioenergetics (Wojtovich et al., 2012). In aged hearts, both endogenous protective mechanisms such as IPC and drugs mimicking IPC are ineffective. For instance, DIAZO and other KATP-activators (effective cardioprotective drugs in young hearts) are poorly effective in aged hearts (Tani et al., 2001; Heinen et al., 2008a,b; Boengler et al., 2009). An exhaustive explanation of this loss of efficacy is missing, although several authors hypothesized that it can be due to channelopathies, linked to defective expression/function of some types of mitoK channel in aged myocardium (Tani et al., 2001; Pi et al., 2007).

In agreement with the literature (Jahangir et al., 2001), aged cardiac mitochondria showed a more depolarized mitochondrial membrane and a lower calcium upload. As expected, we observed a higher vulnerability to I/R injury in elderly animals, likely to be due to possible aging-dependent defects in expression/function of mitoK channels, or with alterations in the signal transduction downstream of mitoK channel activation in aged myocardium (Pi et al., 2007).

As regards mitoBK channels, composed by at least two kinds of (the pore-forming α subunit and a regulatory β1 subunit), data are conflicting (Wang et al., 2008; Singh et al., 2013). Recently, it has been demonstrated that pharmacological targeting of mitoBK channels with a well-known BK opener (NS1619) can induce cardioprotection not only in the young but also in the aged rat heart (Huhn et al., 2012). However, NS1619 is reported as a non-selective agent, and a number of further a specific actions have been demonstrated (Testai et al., 2015). On the other hand, it has been observed that mitoBK-dependent helium’s effects on reduction of infarct size in young rats were lost in aged rats. Indeed, aging-related decrease in density of BK β_1_ subunit expression may justify this loss of cardioprotection, together with downregulation of signaling steps underlying the PKA activation and connexin 43 (Heinen et al., 2008b, 2014).

In this context, the main finding of this study is that NAR, a flavanone abundant in the *Citrus* genus, is endowed with cardioprotective activity, not only in young animals (Testai et al., 2013b), but also in 1 year old ones. Indeed, NAR-treated 1 year old rats showed a minor extension of myocardial damage both in *ex vivo* and in *in vivo* I/R protocols. Noteworthy, cardioprotection mediated by NAR failed when PAX was administered before the NAR treatment, leading us to speculate that mitoBK channels may be involved.

Such a hypothesis has been evaluated in mitochondria isolated from hearts of 1 year old rats, where the flavonoid produced typical effects of mitoK openers: mild depolarization of mitochondrial membrane, reduced accumulation of calcium ions into the matrix and influx of potassium-mimetic thallium ions.

H9c2 cells represent a model of normal primary cardiomyocytes regarding energy metabolism; they have been successfully used as a reliable *in vitro* model to simulate cardiac ischemia–reperfusion injury (Calderone et al., 2010; Kuznetsov et al., 2015) and to study myocardial pathophysiology including aging processes (Spallarossa et al., 2009). Dox treatment of H9c2 cells caused a clear expression of senescence hallmarks: β-galactosidase positive staining, p16 and p21 mRNA increase, cell cycle arrest, and DNA damage (Spallarossa et al., 2009; Bernardes de Jesus and Blasco, 2012). In particular, cell cycle analysis evidenced a cell cycle arrest in G2/M phase, in accordance with previous studies (Vulliamy, 2009; Lüpertz et al., 2010; Oyama et al., 2011; Gire and Dulic, 2015).

In agreement with data obtained in hearts of 1 year old rats, NAR evoked cytoprotective effects even in senescent H9c2 submitted to A/R.

Western Blot analysis allowed us to evaluate the influence of aging on expression of subunits forming mitoBK channel, in heart tissue as well as in H9c2 cardiomyoblasts. In agreement with results obtained in rat mesenteric artery (Shi et al., 2013), mitoBK subunits α and β were reduced in expression, but were still present in cardiac tissues of 1 year old rats as well as in senescent H9c2 cells.

## Conclusion

In aged hearts, a reduced effectiveness of well-known cardioprotective drugs (for instance, the KATP-opener DIAZO) has been observed. In this paper, we first report that NAR, a flavonoid derivative, exhibits cardioprotective effects against I/R injury, even in 1 year old animals. Furthermore, in this paper we demonstrated also a possible correlation between cardioprotection and activation of mitoBK channels, indicating that mitoBK, albeit reduced in expression with aging, may represent a reliable pharmacological target for cardioprotective purposes.

Further studies are needed to better understand the whole pathway involved in the cardioprotection afforded by NAR and other BK activators, and to investigate the promising perspective of a rational nutraceutical use of NAR, aimed to improve mitochondrial function and/or slow down the mitochondrial dysfunction in aging.

## Ethics Statement

It has been approved by the Italian Ministry of Health. All the experimental procedures were carried out following the guidelines of the European Community Council Directive 2010/63EU and have been approved by the Animal Care Committee of the University of Pisa, Italy (Protocol. N. 37321, October 22nd, 2013).

## Author Contributions

LT designed and performed the research, analyzed the data and wrote the manuscript. ED, IP, and AM contributed to the experimental work. LP, CG, MB, AB, and CM contributed to the analysis of the data and the writing of the manuscript. VC designed the research, analyzed the data and contributed to the writing of the manuscript.

## Conflict of Interest Statement

The authors declare that the research was conducted in the absence of any commercial or financial relationships that could be construed as a potential conflict of interest.

## Abbreviations

Ai: infarct area
*A*_LV_: left ventricle area
DIAZO: diazoxide
DMEM: Dulbecco’s modified Eagle’s medium
DMSO: dimethylsulfoxide
Dox: Doxorubicin
dP/dt: the rate of rise of the left ventricular pressure
ECG: electrocardiogram
FBS: fetal bovine serum
HR: heart rate
IM: incubation medium
IPC: ischemic pre-conditioning
I/R: ischemia/reperfusion
LAD: left anterior descending coronary artery
LVDP: left ventricular developed pressure
MI: myocardial infarct
mitoBK: mitochondrial large conductance calcium-activated potassium channel
mitoKATP: mitochondrial ATP-sensitive potassium channels
MPTP: mitochondrial permeability transition pore
NAR: Naringenin
PAX: paxilline
PE50: polypropylene tube
RPP: rate pressure product
TPP^+^Cl^-^: tetraphenylphosphonium chloride
TTC: 2,3,5-triphenyltetrazolium chloride
Ψm: mitochondrial membrane potential.
